# Zuclopenthixol inhibits residual activity of acid sphingomyelinase in Niemann pick disease type B: a case report with in vitro validation

**DOI:** 10.1186/s13023-026-04238-0

**Published:** 2026-04-09

**Authors:** Maximilian Schiller, Katharina Marie Steiner, Udo Bonnet, Erich Gulbins, Norbert Scherbaum

**Affiliations:** 1 Institute of Molecular Biology, University Hospital Essen, University of Duisburg-Essen, Essen, Germany; 2 Department of Psychiatry and Psychotherapy, Medical Faculty, LVR-University Hospital Essen, 45147 Essen, Germany; 3 Department of Psychiatry, Psychotherapy, and Psychosomatic Medicine, Evangelisches Krankenhaus Castrop-Rauxel, Grutholzallee 21, D-44577 Castrop-Rauxel, Germany

**Keywords:** Acid sphingomyelinase, Zuclopenthixol, Niemann-pick disease

## Abstract

Niemann-Pick disease type B (NPD-B) is a rare lysosomal storage disorder characterized by residual activity of acid sphingomyelinase (ASM). While functional inhibitors of ASM (FIASMAs) are widely prescribed as psychotropic medications, they may pose a particular risk to patients with NPD-B by further reducing the already impaired enzymatic function. Here, we report the case of a 20-year-old male with genetically confirmed NPD-B who experienced rapid clinical deterioration following the administration of zuclopenthixol, a drug not previously associated with FIASMA activity. Within 48 hours of treatment initiation, the patient developed profound lethargy and markedly elevated creatine kinase (CK) levels of up to 22,000 U/L, consistent with rhabdomyolysis. Symptoms resolved quickly after discontinuation of zuclopenthixol. In vitro experiments using a radioactive [^1 4^ C]-sphingomyelin assay in Jurkat cells demonstrated that zuclopenthixol dose-dependently inhibited ASM activity by up to 71.5%. Zuclopenthixol had not previously been recognized as a FIASMA and might therefore have been considered a rational choice for treating patients with NPD-B. Our findings challenge this assumption by identifying zuclopenthixol as a potent inhibitor of ASM activity. This novel insight is of high clinical relevance, given the frequent use of antipsychotics in the management of neuropsychiatric symptoms in lysosomal storage disorders. We propose that zuclopenthixol and other potential FIASMAs be carefully re-evaluated for use in this vulnerable patient population.

## Background

Niemann-Pick disease (NPD) is a rare autosomal recessive disorder characterized by lysosomal storage dysfunction. The estimated incidence is approximately 0.4 to 1 per 100,000 live births [1]. The disease is classified into four types. Niemann-Pick types A and B are caused by mutations in the *SMPD1* gene, leading to a deficiency in acid sphingomyelinase (ASM) [2, 3]. Niemann-Pick types C and D are caused by mutations in the *NPC1* and *NPC2* genes, leading to abnormal intracellular cholesterol storage [4].

Patients with NPD type A have an almost complete deficiency of ASM activity, resulting in severe hepatosplenomegaly and rapidly progressive neurodegeneration, which typically leads to death within the first few years of life [5, 6]. In contrast, type B shows reduced but residual sphingomyelinase activity, allowing patients to reach adulthood. While the central nervous system (CNS) is not directly involved, patients often experience hepatosplenomegaly and pulmonary complications [7–9].

Many antipsychotics belong to a class of medications known as functional inhibitors of acid sphingomyelinase (FIASMAs). These drugs partially block ASM activity but allow sufficient metabolism for cellular survival in healthy individuals [10, 11]. Because patients with NPD-B depend entirely on their residual ASM activity, additional pharmacological inhibition may cross a critical metabolic threshold. In this report, we present a case of a patient with Niemann-Pick disease type B in whom treatment with zuclopenthixol, a drug not previously known to inhibit ASM, resulted in a clinical presentation resembling NPD type A. We show that this adverse effect of zuclopenthixol might be caused by drug-induced inhibition of the ASM.

## Case presentation

A 20-year-old male patient was admitted to inpatient psychiatric treatment by his parents due to aggressive behaviour. The patient had been diagnosed with Niemann-Pick disease type B through molecular genetic testing in early childhood, which identified the SMPD1 variant c.1177T > G (p.Trp393Gly). Clinically, he presented with short stature, dystrophy, hepatosplenomegaly, and a known history of intellectual disability. Prior medication included lorazepam, risperidone, and biperiden, all of which were continued without changes. Upon admission, laboratory tests revealed thrombocytopenia with a platelet count of 116 × 10^9^/L, creatine kinase (CK) levels of 196 U/L, aspartate aminotransferase (AST) of 57 U/L, and alanine aminotransferase (ALT) of 72 U/L, with no other significant abnormalities.

Due to the patient’s aggressive and agitated behaviour, treatment with zuclopenthixol was initiated for three days, titrating the dose over three days up to 12 mg per day. Initially, there was a noticeable reduction in aggression, but after two days, the patient developed excessive drowsiness, lethargy, and a general reduction in activity and responsiveness. The patient did not develop fever, muscular rigidity, or focal neurological signs. There were no indications of a disrupted sleep-wake cycle or other signs suggestive of delirium. As these symptoms were evaluated as adverse effects of zuclopenthixol, the medication was discontinued after two days.

**Fig. 1:**
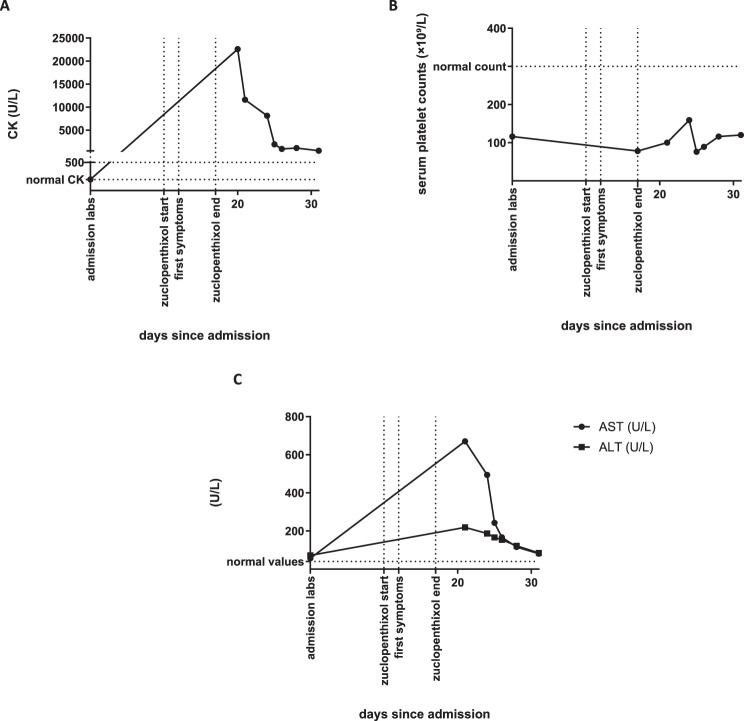
Temporal changes in serum biomarkers in relation to zuclopenthixol treatment. Blood samples were obtained at admission (Day 0, baseline, prior to initiation of zuclopenthixol), during treatment, and during follow-up after discontinuation of zuclopenthixol. The x-axis indicates days since hospital admission. Graph A shows serum creatine kinase levels (CK, U/L) over time in relation to zuclopenthixol treatment. Graph B shows serum platelet counts (×10^9^/L). Graph C shows serum aspartate aminotransferase (AST) and alanine aminotransferase (ALT) levels (U/L) over the same time period. A marked increase in CK and transaminases is observed after drug administration, with peak values during treatment and gradual normalization following discontinuation of zuclopenthixol

Laboratory investigations revealed a massive CK elevation, up to 22,000 U/L, indicating significant muscle injury, potentially rhabdomyolysis. At that time, the patient appeared clinically tired and withdrawn; there were no signs of trauma, prolonged physical restraint, intramuscular injections, dehydration, prolonged immobilization, or sustained extreme physical agitation that could independently explain the CK elevation. Additionally, liver transaminases (AST and ALT) were elevated, and the patient’s platelet count had slightly decreased (Fig. 1). Recovery was gradual, with CK and transaminase levels returning to normal levels over the following three days (Fig. 1). Supportive therapy with intravenous fluids was initiated only three days after zuclopenthixol discontinuation. The patient’s clinical condition improved progressively, although symptoms such as psychomotor slowing persisted for an additional two days after zuclopenthixol was discontinued. After full recovery, the patient was admitted to a specialized clinic in order to evaluate a therapy with recombinant acid sphingomyelinase (olipudase alfa).

## Methods

### Clinical data collection and laboratory analysis

Clinical and laboratory parameters were systematically recorded before, during, and after the administration of zuclopenthixol. Laboratory values were obtained from venous blood samples using standard automated analyzers at the hospital’s central laboratory. Clinical observations, including neurological status, level of sedation, and muscular symptoms, were documented throughout treatment.

### Cell culture and treatment

Jurkat cells were grown in RPMI-1640 supplemented with 10 mM HEPES, 2 mM L-glutamine, 1 mM sodium pyruvate, 100 µM nonessential amino acids, 100 U/mL penicillin, 100 µg/mL streptomycin and 10% fetal calf serum.

Jurkat cells served as model cells. Jurkat cells were cultured at 250,000 cells/mL and incubated with 1 µM, 2.5 µM or 5 µM zuclopenthixol for 24 h or left untreated. Zuclopenthixol was dissolved in H_2_O at 10 mM and then diluted into the samples.

Before performing the ASM assay, cell numbers and morphology were assessed to exclude overt cytotoxic effects. No reduction in cell numbers or morphological alterations indicative of cytotoxicity were observed under any of the conditions tested.

Cells were collected and washed twice in HEPES/Saline (H/S; 132 mM NaCl, 20 mM HEPES [pH 7.4], 5 mM KCl, 1 mM CaCl_2_, 0.7 mM MgCl_2_, 0.8 mM MgSO_4_). Cells were lysed in 250 mM sodium acetate (pH 5.0) and 0.2% NP40 for 5 min and sonicated in a tip sonicator twice for each 10 sec to achieve complete cell disruption. The enzyme assay was initiated by addition of 0.05 µCi [^14^C]sphingomyelin (52 mCi/mmol; ARC) to the samples. To this end, the substrate [^14^C]sphingomyelin was dried for 10 min in a SpeedVac, resuspended in 250 mM sodium acetate (pH 5.0) and 0.1% NP40, and sonicated for 10 min in a bath sonicator prior to use. After addition of the substrate, the samples were incubated for 30 min at 37 °C with shaking at 300 rpm, extracted in 4 volumes of CHCl_3_:CH_3_OH (2:1, v/v), centrifuged to get phase separation, and an aliquot of the upper aqueous phase was scintillation-counted to determine the release of [^14^C]phosphorylcholine from [^14^C]sphingomyelin.

All values were normalized to cell numbers and are given as pmol sphingomyelin/h/10^6^ cells, which is the standard normalization approach in ASM/FIASMA assays and ensures comparability across conditions. Each condition was analyzed in four independent biological replicates (*n* = 4), each performed on separately cultured cell batches. Each biological replicate was measured once according to standard protocol for the radiolabeled ASM assay

Cell viability was assessed after 24 hours of incubation using trypan blue exclusion. Jurkat cells were incubated with 1 µM, 2.5 µM, or 5 µM zuclopenthixol for 24 h or left untreated as control. For each condition, at least 100 cells per quadrant were counted in all four quadrants of a Neubauer counting chamber. Dead cells were identified as trypan blue–positive. The percentage of cell death was calculated as the number of trypan blue–positive cells divided by the total number of counted cells.

### Statistical analyses

Statistical analyses were performed using GraphPad Prism 9 (GraphPad Software, San Diego, USA). Data are presented as mean ± standard deviation (SD). Comparisons between groups were conducted using a one-way ANOVA followed by Bonferroni’s multiple comparison test. A p-value < 0.05 was considered statistically significant.

## Results

**Fig. 2:**
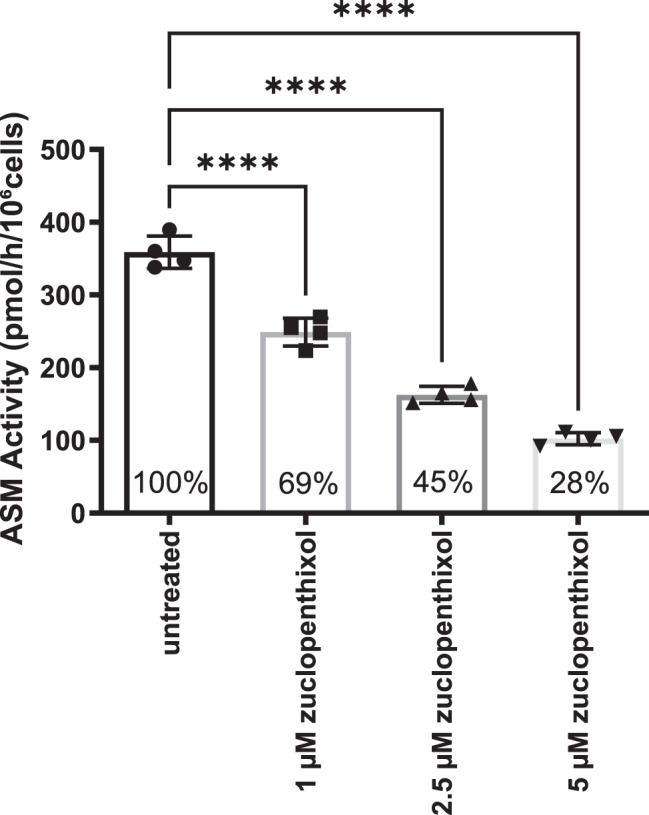
Dose-dependent inhibition of acid sphingomyelinase (ASM) activity by zuclopenthixol in Jurkat cells. Jurkat cells were incubated for 24 hours with 1 μM, 2.5 μM, or 5 μM zuclopenthixol, or left untreated as control. Bars represent mean ASM activity (pmol sphingomyelin hydrolyzed per hour per 10^6^ cells) ± SD from four independent experiments. Individual data points are shown for each condition. Percent residual ASM activity relative to untreated control (set at 100%) is indicated below each bar. A significant, dose-dependent reduction in ASM activity is observed across all concentrations (****p < 0.0001; one-way ANOVA)

To further study the influence of zuclopenthixol on acid sphingomyelinase (ASM), we performed an in vitro study using Jurkat cells (Fig. 2), since these cells are a well-known cell line to study ceramides and lipid metabolism on in general [12]. Incubation with 1 µM, 2.5 µM or 5 µM zuclopenthixol for 24 hours resulted in a significant inhibition of ASM activity.

Specifically, ASM activity decreased from 358.9 ± 21.6 pmol sphingomyelin/h/10^6^ cells in untreated cells to 248.9 ± 19.7 pmol sphingomyelin/h/10^6^ cells at 1 µM zuclopenthixol, 162.8 ± 12.3 pmol sphingomyelin/h/10^6^ cells at 2.5 µM, and 102.3 ± 8.5 pmol sphingomyelin/h/10^6^ cells at 5 µM. This corresponds to a 30.6% inhibition at 1 µM zuclopenthixol, 54.6% inhibition at 2.5 µM, and 71.5% inhibition at 5 µM compared to untreated cells.

Trypan blue exclusion after 24 h showed consistently low cell death rates in all conditions (untreated: 2–4%; 1 µM: 1–3%; 2.5 µM: 1–5%; 5 µM: 2–4%), with no relevant differences between treated and untreated cells, indicating absence of nonspecific cytotoxicity.

These findings indicate a dose-dependent inhibition of ASM activity by zuclopenthixol.

## Discussion

Since zuclopenthixol is not yet known as FIASMA, the metabolic deterioration of the patient described was unexpected. However, in this case, the patient’s laboratory results, particularly the massive CK increase along with clinical signs of lethargy and impaired activity, strongly suggest that zuclopenthixol might have had an inhibitory effect on ASM activity.

When evaluating the acute metabolic deterioration and CK elevation, malignant neuroleptic syndrome (MNS) must be considered. According to DSM-5 and established clinical diagnostic frameworks, MNS is characterized by a combination of core and supportive features. The core features include hyperthermia, severe muscle rigidity, and elevated CK levels in the context of recent dopamine antagonist exposure. Supportive features include alterations in mental status, autonomic instability (tachycardia, blood pressure fluctuations, diaphoresis), leukocytosis, and frequently acute renal impairment [13].

In our patient, however, several of these defining clinical features were absent. Despite the pronounced CK elevation, the patient showed no fever, no muscular rigidity, no autonomic instability, no leukocytosis, and no signs of renal impairment, making the clinical syndrome atypical for MNS. The temporal evolution also differed markedly from the expected MNS pattern. While MNS typically evolves over several days and recovery—even after cessation of the neuroleptic—usually requires 10–30 days, our patient exhibited a rapid decline in CK values and a marked clinical improvement within only three days after stopping zuclopenthixol [14]. This unusually fast resolution, combined with the absence of cardinal diagnostic criteria, makes MNS an unlikely explanation for the observed deterioration.

In light of the clinical presentation and laboratory findings, we considered the symptoms to be most consistent with a pharmacologically induced reduction of residual ASM activity due to zuclopenthixol.

To further investigate this potential mechanism, we conducted an in vitro study. Incubation of cells with zuclopenthixol for 24 hours resulted in a significant, dose-dependent inhibition of ASM activity of up to 71.5% compared to untreated cells. ASM is normally bound to intralysosomal membranes, which protects it from proteolytic inactivation [15]. It is possible that zuclopenthixol, as a lipophilic weak base, accumulates in lysosomes and disrupts the association of ASM with these lysosomal membranes, leading to its proteolytic degradation [16]. This mechanism could explain the drug’s unexpected effects in this patient [11, 16].

While this mechanism is consistent with established models of FIASMA activity, it remains speculative for zuclopenthixol, as lysosomal pH changes or direct destabilization of ASM have not been experimentally demonstrated for this compound. Therefore, further studies are required to confirm whether zuclopenthixol induces lysosomal alterations comparable to those reported for other cationic amphiphilic drugs.

In clinical maintenance therapy, plasma concentrations of zuclopenthixol typically range from 5.9 to 9.5 nmol/L, corresponding to low-nanomolar extracellular exposure [17]. However, due to pronounced intracellular and lysosomal accumulation of cationic amphiphilic drugs, intracellular concentrations are expected to markedly exceed plasma levels [18]. Therefore, concentrations up to 5 µM were used in our in vitro experiments to approximate the higher lysosomal exposure expected in vivo and to allow comparison with established FIASMA assays.

Zuclopenthixol is also metabolized in part by CYP2D6, and genetic polymorphisms can influence plasma concentrations [17, 19, 20]. Pharmacogenetic testing was not available in this retrospective case; however, the patient did not exhibit clinical features typically associated with excessive systemic exposure, and the rapid improvement after discontinuation argues against prolonged accumulation due to impaired CYP2D6 metabolism.

Although zuclopenthixol has not previously been evaluated for FIASMA activity, flupentixol—another thioxanthene derivative—has been shown to inhibit ASM [11]. This makes an ASM-inhibiting effect of zuclopenthixol pharmacologically plausible, although no data were available prior to this case.

Various experimental methods exist to assess FIASMA activity, but such assays are not part of standard drug development and are not required by regulatory agencies.

Most psychotropic drugs have already been screened for FIASMA activity, and such effects are not harmful in individuals with normal ASM function—indeed, they may contribute to the antidepressant mechanism of several medications [21]. Therefore, routine ASM testing or additional psychotropic screening is not indicated. The relevance of ASM inhibition becomes clinically meaningful mainly in patients with markedly reduced residual ASM activity, such as those with Niemann-Pick disease.

The observed clinical picture closely resembles that of Niemann-Pick disease type A with nearly absent ASM activity resulting in severe cellular dysfunction. Given these findings, it is advisable to avoid the use of zuclopenthixol in patients with Niemann-Pick disease, particularly those with type B.

## Conclusion

In summary, we describe a patient with Niemann-Pick disease type B who experienced acute metabolic deterioration after treatment with zuclopenthixol. Our clinical observations, supported by in vitro data, identify zuclopenthixol as a previously unrecognized inhibitor of acid sphingomyelinase. While this finding does not affect routine psychiatric practice, it is clinically relevant for individuals with markedly reduced residual ASM activity. In such cases, unrecognized FIASMA properties of psychotropic medications may pose a risk for metabolic decompensation. Clinicians should therefore exercise caution when prescribing antipsychotic agents to patients with Niemann-Pick disease, particularly type B, in whom even partial inhibition of ASM may have significant consequences. Further studies are warranted to better characterize the FIASMA potential of commonly used psychotropic drugs in this specific patient population.

## Limitations

This report has several limitations. It describes a single patient, and the mechanistic conclusions are supported by in vitro data obtained from a non-disease-specific cell line. Functional confirmation in primary cells from patients with Niemann-Pick disease was not performed. Therefore, the findings should be interpreted with caution and warrant further investigation.

## Data Availability

All data generated or analysed during this study are included in this published article.
